# Surface Treatments of Coffee Husk Fiber Waste for Effective Incorporation into Polymer Biocomposites

**DOI:** 10.3390/polym13193428

**Published:** 2021-10-07

**Authors:** Bárbara Maria Mateus Gonçalves, Mayara de Oliveira Camillo, Michel Picanço Oliveira, Lilian Gasparelli Carreira, Jordão Cabral Moulin, Humberto Fantuzzi Neto, Bárbara Ferreira de Oliveira, Artur Camposo Pereira, Sergio Neves Monteiro

**Affiliations:** 1 Forest and Wood Sciences Department, Federal University of Espírito Santo, Jeronimo Monteiro, Vitória 29550-000, Brazil; barbarammateus@hotmail.com (B.M.M.G.); maycamillo@gmail.com (M.d.O.C.); michelpicanco@gmail.com (M.P.O.); jordao_cm@hotmail.com (J.C.M.); hfantuzzi@yahoo.com.br (H.F.N.); 2 Rural Engineering Department, Federal University of Espírito Santo, Alto Universitário, sn., Alegre 29500-000, Brazil; lcarreira83@gmail.com; 3 Advanced Materials Department, Darcy Ribeiro Northern Fluminense State University, Campos dos Goytacazes 28013-602, Brazil; barbara.fo@gmail.com; 4 Military Institute of Engineering—IME, Materials Science Program, Praça General Tibúrcio 80, Urca, Rio de Janeiro 22290-270, Brazil; camposo.artur@gmail.com

**Keywords:** coffee husk fiber, fiber surface, polymer biocomposite

## Abstract

Natural lignocellulose fibers have been extensively investigated and applied as a reinforcement of polymer composites in industrial applications from food packing to automotive parts. Among the advantages of natural fibers stands their relatively low cost and sustainable characteristics. These are accentuated in the case of residual fibers such as those obtained from coffee husks, an agribusiness waste, usually burnt or disposed into the environment. As composite reinforcement, hydrophilic natural fibers display adhesion problems to the most hydrophobic polymer matrices. This adhesion might be improved with distinct types of fibers surface treatments. In the present work, the effectiveness of three surface treatments applied to coffee husk fiber wastes (CHFW) were investigated, aiming to improve the tensile performance of castor oil-based polyurethane (COPU) biocomposites. The effects of treatments associated with (i) chemical with sodium hydroxide, (ii) physical by temperature and pressure and hydrothermic treatment, and (iii) biological by fermentation with *Phanerochaete Chrysosporium* fungus were evaluated by means of Fourier transformed infrared spectroscopy, X-ray diffraction, thermal analyses and morphology by scanning electron microscopy for different concentration of NaOH, different hydrothermic times at 121 °C/98 kPa and exposition to *P. chrysosporium*. The most effective treatment was the hydrothermal one at 121 °C and 98.06 kPa for 30 min. Preliminary tensile tests were performed in COPU biocomposites reinforced with 20% CHFWs subjected to the optimized conditions for each distinct type of treatment. The results indicated that the hydrothermal treatment promoted significant enhancement in the fiber/matrix interfacial bond, increasing the tensile strength up to 60% compared to COPU reinforced with in natura CHFWs fibers. It is important to mention that these composites can be applied as plastic wood for household items’ internal parts and in the automobile industry.

## 1. Introduction

The environmental awareness movement has motivated a quest for the use of alternative renewable resources. Numerous studies report the use of vegetable fibers, also known as natural lignocellulosic fibers (NLF), as reinforcing fillers in polymeric composites [1,2,3,4]. NLF wastes such as pineapple leaves, banana stem, sugarcane bagasse, and coffee husk are commonly used fibers [3,4] that provide higher amounts of cellulose, a semicrystalline, rigid and resistant polysaccharide [5,6,7].

Brazil is the world’s largest exporter of coffee. The National Supply Company—CONAB—forecasts a production of approximately 49 million bags for the 2021 harvest [6]. It is one of the most popular beverages in the world, and approximately 50% of its weight is discarded during the fruit’s processing. Furthermore, coffee is pointed out as a promising reusable source of agricultural residues [7,8,9].

Different combinations between polymeric matrices and coffee husk fibers were recently tested [10,11,12]. Collazo-Bigliardi et al. [10] reported a 148% increase in the elastic modulus of hybrid composites of poly(lactic acid) and cornstarch reinforced with 1% cellulose nanocrystals extracted from coffee husks. Huang et al. [11] obtained improved tensile strength (44.51 MPa) and flexural strength (69.58 MPa) for high-density polyethylene composites incorporating 70% of these fibers. Borghesi et al. [12] related that the addition of coffee husks as reinforcement to a polycaprolactone matrix increased biodegradation rates and reduced the total cost of the material produced.

Using NLFs has numerous advantages. In addition to the composites’ enhanced mechanical properties, they are biodegradable, noncorrosive, lightweight, inexpensive, abundant, easy to process, have high strength, and good thermal properties [8,9]. Although their hydrophilic nature can compromise the properties of the composite owing to their low matrix compatibility and water absorption tendency [8,13,14,15], such disadvantages do not limit their composite use. Indeed, the compatibility between the matrix and reinforcement can be improved through several surface treatments, chemical, physical, or biological, applied to these fibers. Table 1 shows some treatments of natural fiber for applications as reinforcement of biocomposites [15].

The alkaline mercerization technique stands out among the chemical treatments. Indeed, sodium hydroxide (NaOH) solutions trigger the ionization of the hydroxyl groups in the medium, chemically modifying the fiber structure by breaking the hydrogen bonds [12,13]. This method’s efficiency depends on factors such as reaction time, temperature, and the alkaline solution concentration. However, high concentrations of NaOH solution can intensify fiber delignification, weakening and damaging the NFL microstructure [13,14,15]. 

Physical treatments modify the fibers’ structure without changing their chemical composition. Hydrothermal treatments, such as self-hydrolysis, are examples of this method. Autoclave- or reactor-increased temperatures raise steam pressure, which then dissolves part of the material’s hemicellulose and extractives content. 

Despite NFLs’ wide use and positive results, chemical and physical treatments have the following shortcomings: (i) risky and expensive reagents; (ii) generation of effluents; and (iii) equipment’s high energy consumption. Therefore, biological agent techniques become a promising alternative to other treatments [12]. 

Under specific medium conditions, fungi and enzymes can be used to degrade lignin and hemicellulose and remove part of the extractives through enzymatic hydrolysis reactions [16]. So, the fungal attack on fibers occurs through enzymes produced by these microorganisms. The popularly known white rot fungi, for example, are considered a primary lignin degrader, which produce oxidative enzymes, such as peroxidases and laccases [17]. 

**Figure 1:**
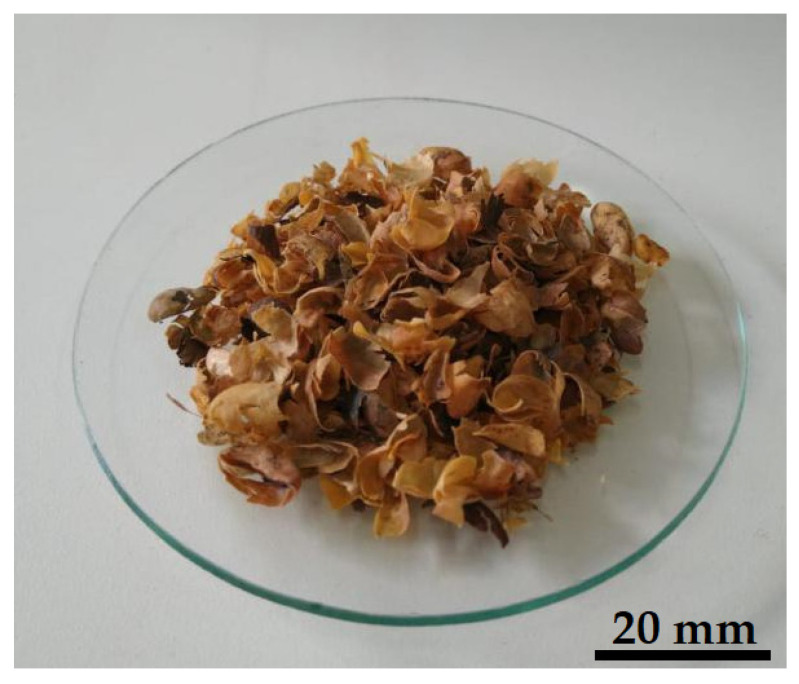
Coffee husk fiber waste (CHFW).

In this study, the influence of different chemical, physical, and biological treatments applied on coffee husk fiber wastes (CHFW), as illustrated in Figure 1, was evaluated. The main difference of this work is the study of the relationship between physical, biological, and chemical treatment with chemical, morphological, and thermal natural fiber properties. In here, it is investigated which one offers a higher cellulose molecule exposure, thereby unleashing the full potential of CHFW as an effective a polymeric biocomposite reinforcing filler to be incorporated into castor-oil-based polyurethane (COPU) matrices. These biocomposites can be applied, for example, as plastic wood in household items and automobile interior parts.

## 2. Materials and Methods

### 2.1. Materials

The coffee husk fiber waste (CHFW) was supplied, as cost-free residue from the 2019 crop, by the coffee producer Ricardo Potratz located in the city of Alegre, state of Espírito Santo, Brazil, shown in Figure 1. The as-received CHFW particles with size varying from 3 to 5 mm were cleaned in running water and then dried for a couple of hours in a stove at 50 °C until constant weight. 

The castor-oil-based polyurethane (COPU) was synthesized from polyol mixed with diphenylmethane diisocyanate, both commercially supplied by Imperveg Polímeros Indústria e Comércio LTDA, São Paulo, Brazil. The synthesis of COPU was performed just before incorporating the CHFW for composite fabrication, following the process further indicated in Section 2.2.3.

### 2.2. Methods 

#### 2.2.1. Treatments of CHFWs

##### Chemical Treatment: Mercerization

To evaluate the influence of alkaline concentration on the CHFW surface treatment, three solutions of sodium hydroxide (NaOH) were prepared at concentrations of 5%, 10%, and 20% (% mass/volume) named NaOH 5, NaOH 10, and NaOH 20, respectively. These concentrations were chosen by a previous study that demonstrated that high NaOH concentrations degraded fibers. The proportion of CHFW and alkaline solution used was 1:10 (% mass/volume), the contact time, reaction temperature, and medium agitation remained constant during 2 h, at 70 °C, and 500 rpm, respectively. At the end of this procedure, the fibers were first vacuum filtered and washed with distilled water until the filtrate had a neutral pH. Then they were oven-dried at 50 °C over 24 h to reach a uniform weight.

##### Physical Treatment: Hydrothermal

CHFW used in a proportion of 1:20 (% mass/volume) was immersed in distilled water using a 500-mL Erlenmeyer flask. This treatment evaluated the exposure of cellulose molecules with regard to time. The containers were conditioned in a vertical autoclave Phoenix Luferco AV-100 (Araraquara, Brazil) programmed to operate at 121 °C and 98 kPa for the following intervals: 30, 60, and 120 min, named HYD 30, HYD 60, and HYD 120, respectively. After this step, the fibers were vacuum filtered and oven-dried at 50 °C for 24 h to reach a uniform weight.

##### Biological Treatment: Solid-State Fermentation by Phanerochaete Chrysosporium

Solid-state fermentation was conducted with the use of *Phanerochaete chrysosporium*. The fungus was inoculated in the CHFWs to verify the degree of exposure of the cellulose fibers caused by this microorganism, and 10 g CHFW was added in 8–500 mL distinct Erlenmeyer flasks, previously sterilized in a vertical autoclave. 

The fermentations took place in a laminar flow hood with insertion 0.1 g of inoculum from a solid stock, with a composition of 20:80 (wheat bran: cane bagasse) into each Erlenmeyer flask. To achieve the medium’s ideal moisture content (60%), 15 mL of a saline solution with the composition described by Urbánszki et al. [18]. 

Every 24 h after the fermentations, two flasks were removed and placed in an autoclave for 30 min to ensure cell death. The material was then vacuum filtered, washed with 250 mL distilled water, and finally oven-dried at 50 °C until the sample showed no mass variation. This treatment was performed at 4 different times, 24, 48, 72, and 96 h, which were identified as BIO 24, BIO 48, BIO 72, and BIO 96, respectively.

##### Chemical Characterization

To determine the content of lignocellulosic materials in fresh CHFW after the treatments, the following analyses were conducted sequentially. Determination of extractive content according to NBR 14853 [19], using: (i) a solution of toluene and ethanol as solvent (2:1) in the first 5 h of extraction; (ii) only ethanol for the second extraction with same duration; and (iii) third extraction conducted at 100 °C in water immersion. With the material free of extractives, the lignin content was calculated according to NBR 7989 [20]. Cellulose and hemicellulose were quantified according to the procedures described by Rowell [21]. The samples’ ash contents were determined based on the TAPPI standard (T 211 om-02) [22]. 

#### 2.2.2. Characterization of CHFWs before and after Treatments

##### True Density

The true density was measured by pycnometry technique using acetone PA (99.5%, NEON) as a fluid. Then, 1.00 g of CHFW was added to 25 mL in the pycnometers. The pycnometers were completely filled, and the mean value of density obtained according to the following equation:

(1) $$\rho_{R}=\left[\frac{M_{A}}{(M_{4}+M_{1})-\left(M_{1}-M_{3}\right)}\right]\times \left(\frac{M_{4}-M_{1}}{V}\right)$$

where $\rho_{R}$ is the true density; M_A_ is the sample mass; M_1_ is the pycnometer mass; M_2_ is the mass of the pycnometer plus the mass of the sample; M_3_ is the mass of the pycnometer containing sample and acetone; M_4_ is the mass of the pycnometer filled with only acetone; and V the pycnometer volume.

##### Fourier Transformed Infrared (FTIR) Spectroscopy

The identification of the fibers’ chemical components in fresh and treated CHFW was done using Fourier transform infrared (FTIR) spectroscopy. The spectra were obtained with a Bruker spectrophotometer, model Tensor 27 (Billerica, MA, USA), using the KBr pellet technique with mid-infrared region scans (4000–400 cm^−1^). 

##### X-ray Diffraction and Morphological Characterization

X-ray diffraction pattern was recorded using Rigaku MiniFlex 600 Diffractometer (Tokyo, Japan) with Cu Kα radiation (λ = 1.54 Å). The samples were scanned at range of 2θ from 5° to 70° with a step size and scan rate of 0.05° and 2° min^−1^, respectively. The main crystalline peaks were identified using a Match software, and the (Ic) was calculated using the Segal method according to Equation (2) [23]:

(2) $$I_{c}=\left(\frac{I_{\left(200\right)}-I_{\mathrm{AM}}}{I_{200}}\right)\times 100\%$$

where I_(200)_ is the maximum intensity of the principal peak with crystallographic plane (2 0 0), which refers to the fraction of crystalline cellulose in the material; IAM is the intensity attributed to 2θ = 18° that represents amorphous cellulose. The CHFW surfaces were observed using scanning electron microscopy (SEM) in secondary electrons in a model JEOL, JSM-IT200 (Tokyo, Japan). Samples were previously coated with gold.

##### Thermal Analyses

Thermogravimetric analysis (TGA), derivative thermogravimetry (DTG), and differential scanning calorimetry (DSC), were carried out using a LabSys Evo Thermogravimetric Analyzer (Caluire, France) from room temperature to 700 °C with a heating rate of 10 °C min^−1^ under inert nitrogen atmosphere to evaluate the thermal degradation of CHFWs

#### 2.2.3. Manufacturing of Composites Reinforced with CHFW

CHFW/COPU composites were manufactured with 20 wt.% of reinforcement. First, 46% of prepolymer synthetized from castor-oil-based polyol with diphenylmethane diisocyanate (MDI) were manually mixed with 54% of polyol, also derived from castor oil, for 2 min and placed in a desiccator coupled with a pump vacuum for 1 min. Previously, these components were oven-dried for 1 h at 100 °C to reduce the moisture content of the resins. After mixing, a suitable amount of CHFW was added to obtain 20 wt.% in the composite. This process was made for NaOH 5, BIO 24, HYD 30 and in natura CHFW.

The mixture formed was poured into a silicone mold with shapes and dimensions defined by ASTM D3039 [24] and then placed in a desiccator for 3 min to induce the formation of bubbles. Finally, the mold was placed in a compressed air reactor at 90 kPa for 72 h.

#### 2.2.4. Mechanical Test Composites 

**Figure 2:**
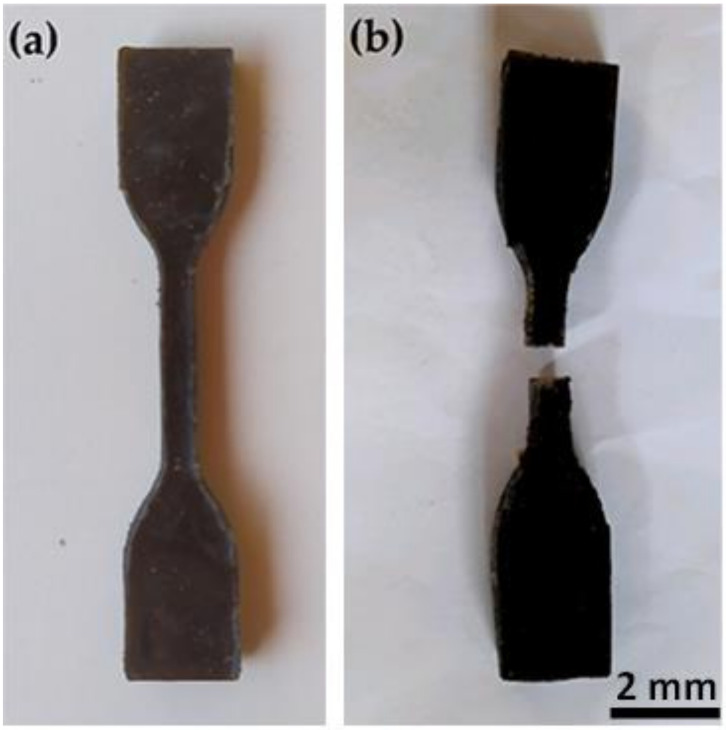
Standard specimen for the tensile test; (**a**) before test and (**b**) after test.

Preliminary tensile tests were conducted at room temperature (RT) on five standard [24] specimens for each treatments, NaOH 5, BIO 30, HYD 30, and in natura with 20 wt.% CHFW composition in a model AME 5KN Oswaldo Filizola universal machine (São Paulo, Brazil), operating with cross-head speed of 1 mm/min until the specimen ruptured using a 2 kN load cell. Figure 2 shows the standard specimens (a) before and (b) after tensile testing. 

## 3. Results and Discussion

### 3.1. Lignocellulosic Characterization and Density of in Natura and Treated Fibers 

The chemical and physical characterizations of plant fibers are crucial in understanding these materials’ relationship with the thermal and mechanical properties of polymer composites. Table 2 shows the values for cellulose, hemicellulose, lignin, extractives, and ashes found in the CHFW after treatments, as well as their density.

The NLFs do not have a fixed composition: their chemical components and corresponding content can vary according to the species, soil conditions, and regional climate [25]. In this study, 20,43% of fresh CHFW extractives were found as 20.43%. Setter et al. [26], Carvalho et al. [27], and Collazo-Bigliardi at al. [28] used fibers from coffee husks in their studies. They obtained 20.53, 6.70, and 17.80% for that very same component, respectively, which validates the present result.

Hemicellulose and lignin play an equally important role in vegetable fibers. Lignin molecules provide resistance, impermeability, and rigidity to cells. Hemicelluloses, however, act as an intermediary between cellulose and lignin bonds, being responsible for the growth and development of fibers [3,4,5]. The percentages identified for cellulose, hemicellulose, and lignin for the in natura CHFW were 30.4, 28.5, and 22.2%, respectively. Similar lignin values were reported by Setter et al. [26] (27.14%) and Huang et al. [11] (20.70%), as well as for cellulose by Collazo-Bigliardi et al. [28] (35.40%).

The beneftis of cellulose’s high degree of polymerization and semicrystalline structure for increasing composites’ stiffness and mechanical strength when added to the polymer matrix are well known [1,2,3,4]. However, access to these molecules is difficult, mainly owing to the hemicelluloses, lignin, and extractives. The treatments were previously applied to degrade the CHFWs as a way to facilitate the exposure of their molecules [4,13]. The increase in cellulose contents shown in Table 1 confirm that all performed treatments achieved this purpose, indicating that structural or chemical changes have occurred and that the fiber/matrix compatibility can be improved [3].

During mercerization, vegetable fibers are ionized by converting the hydroxyl groups into alkoxides [14]. Among all the treatments performed, the NaOH 20 treatment shows the highest percentage of cellulose exposure, 52.8%. Other authors have also obtained a high cellulose molecule exposure rate in fibers treated with NaOH, using natural fiber wastes to incorporate into the polypropylene matrix. Oliveira and Gonçalves [29] reached 66.39% with 2.5% NaOH at 120 °C for 10 min. Bartos et al. [30] reported a cellulose increase higher than 50% when sugarcane bagasse was treated with 15% NaOH for 1 h. Pillai et al. [31] functionalized the banana tree penducles with 5% NaOH at RT (25 °C) for 1 h and obtained an incidence of 79.13% in cellulose.

Among all hydrothermal treatments, HYD 30 exhibited the highest percentage of cellulose exposure (35.1%). Aguilar et al. [32] and Nitsos et al. [33] conducted experiments under different temperature and time conditions, reporting exposures of 65.87 and 32.58% for agave bagasse fibers and almond shell, respectively. Given the longer exposure time of CHFWs during the HYD 60 and 120 treatments, the cellulose molecules may have been degraded, which would explain their decreased content.

On evaluating the contact time of the *P. chrysosporium* fungus with the CHFW, a maximum exposure of cellulose molecules of 38.8% was observed on the second day (BIO 48). Jayapriya and Vigneswaran [34] reported a degradation of jute fiber molecules using the same fungal activity in 30 days. Youssef et al. [35] showed 79% exposure in citrus tree shaving fibers treated for 7 days with *Aspergillus flavus.* Increased tensile strength and hardness of high-density polyethylene (HDPE) composites were observed for 20% incorporation of these fibers into the HDPE matrix. 

In all treatments performed, the lower extractives and ash content can be explained by their low molecular weight, which eases their elimination [4]. Increasing lignin and hemicellulose contents for HYD’s and BIO’s treatments were related to such reduction. A lower hemicellulose percentage was observed in all chemical treatment conditions, indicating that the alkaline medium potentiates the removal of HDPE [23]. 

The observed density variations are also associated with the elimination of substances such as ash and extractives. An inherent characteristic of vegetable fibers is their low density, which affords energy savings when used in automotive parts. For example, abaca fiber with an average 1.5 g·cm^−3^ density proved innovative when used in Daimler–Benz automobile polypropylene-based floors. In fact, its high tensile strength, bending and degradation properties make them comparable to glass fibers. Other automotive companies, such as Audi, BMW, Volkswagen, Toyota, and Ford, use various vegetable fibers in their seat backs, interior doors, and in noise-insulating panels, instrument panel support, and even as engine insulation and internal cover [36].

### 3.2. Fourier Transformed Infrared (FTIR) Spectroscopy

**Figure 3:**
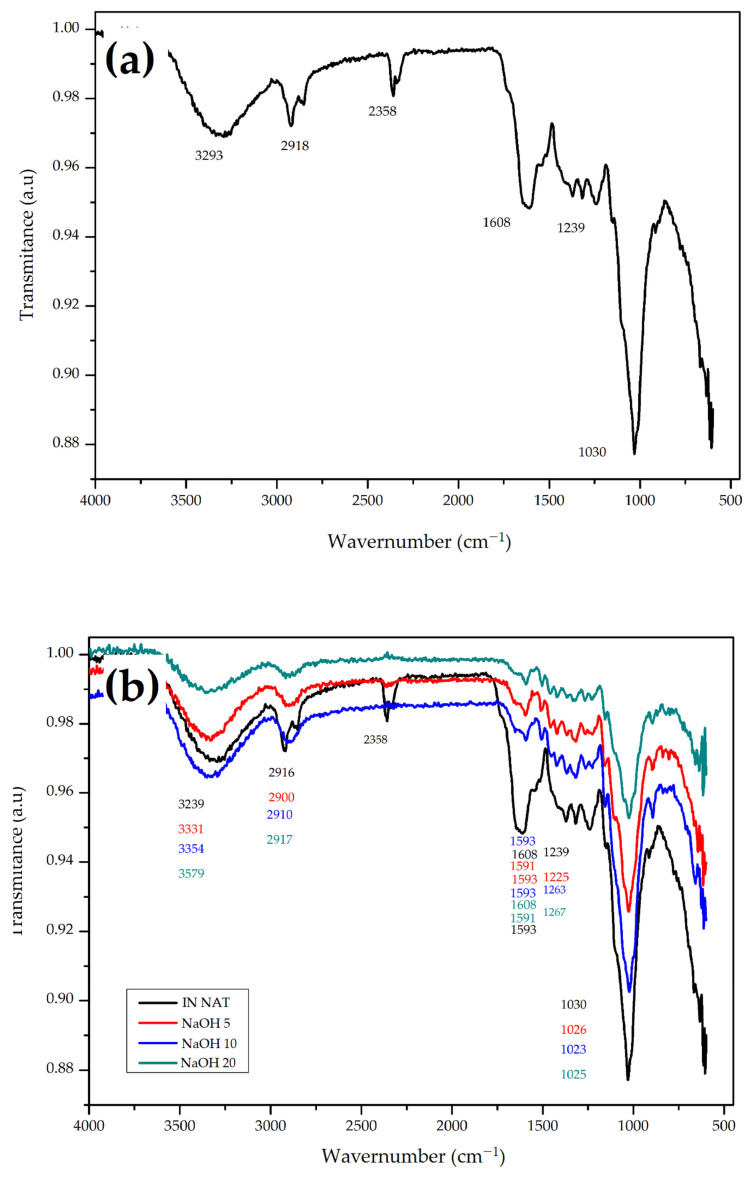

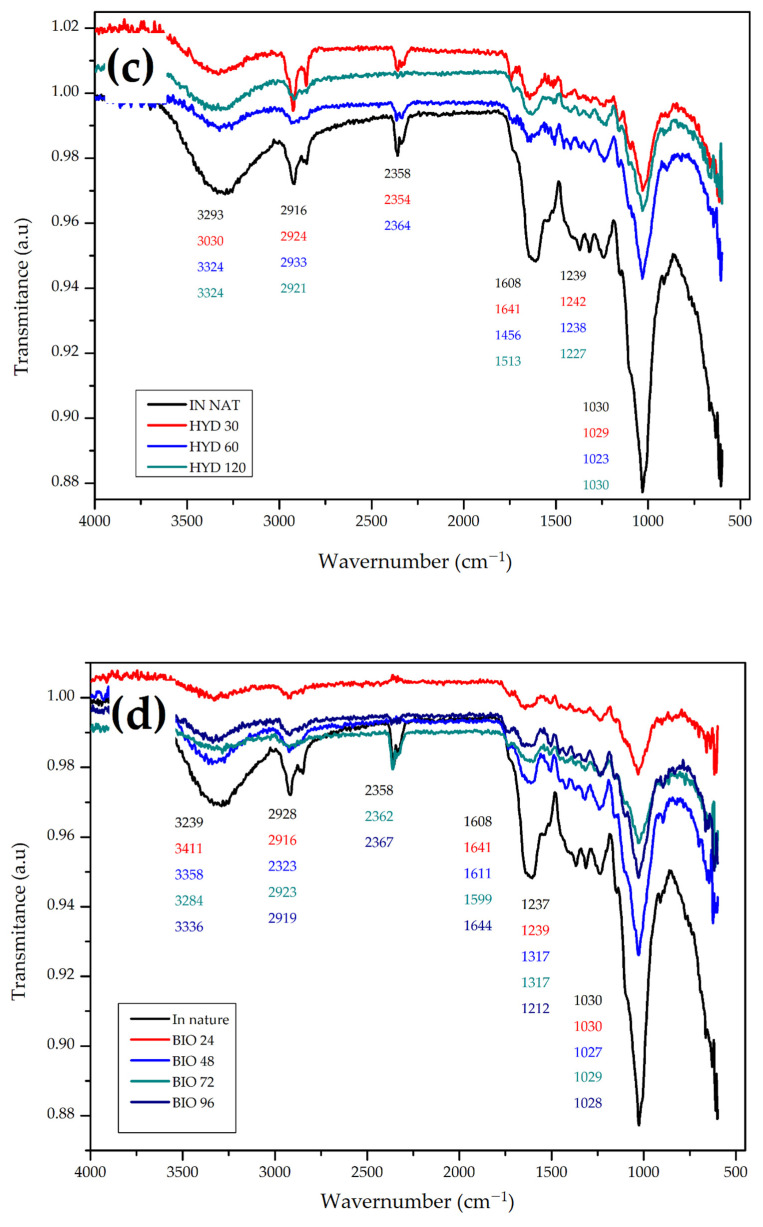
FTIR spectrum of (**a**) in natura CHFW; (**b**) NaOH; (**c**) HYDRO; and (**d**) BIO treatments.

A more detailed analysis of the CHFW composition was obtained using FTIR analysis. Figure 3 shows the obtained spectra. The spectrum presented in Figure 3a provides information about the in natura CHFW. The intense absorption detected at 3293 cm^−1^ indicates hydroxyl groups (OH) associated with the lignocellulosic material, as well as of extractives in the fibers. The second band of absorption, between 2916 and 2358 cm^−1^, represents the aliphatic and alkyl groups of cellulose and the methyl in hemicellulose and in lignin methoxy. The aromatic compounds in lignin were identified by stretching the C=C double bond by 1608 cm^−1^. The band detected at 1370 cm^−1^ is characteristic of the C–OH bond elongation attributed to crystalline cellulose. The absorption bands between 1318 and 1239 cm^−1^ can be attributed to carboxylic acids, acetyl groups, and esters of the material. The intense absorption band at 1030 cm^−1^ is caused by CO bonds stretching and by the deformation of alkoxide groups present in cellulose and ester groups present in lignin [11,16,26]. 

The analysis of the FTIR spectra generated after all treatments (Figure 3b–d) revealed changes in some wavelengths. Generally, for all tested treatments, the extractive compounds’ removal and dissolution of hemicellulose are reflected by changes in the second 2928 cm^−1^ and third 2358 cm^−1^ absorption bands. Mercerization treatments in Figure 3b show no absorbance for values converging to 2358 cm^−1^, thereby proving the effectiveness of the method for removing hemicelluloses, as noticed in Table 1 [23,27,35,37,38].

Changes observed in the fourth absorption band of 1608 cm^−1^ are related to an increased lignin content in all materials after the treatments [39]. In all treatments there was a greater exposure of cellulose molecules associate with the increased values of the first band, and the small change in the sixth absorbance band values are assigned to the CO and OH bonds stretching in the coffee husk’s cellulose [11]. 

### 3.3. X-ray Diffraction (XRD)

It is known that cellulose, unlike hemicellulose and lignin, has a semicrystalline structure due to the interactions of hydrogen bonds and van der Waals forces between its constituent molecules [40]. Specifically for this study, it would be possible to recognize naturally occurring type I pulps as well as type II pulps obtained by mercerization or solubilization treatments [41].

**Figure 4:**
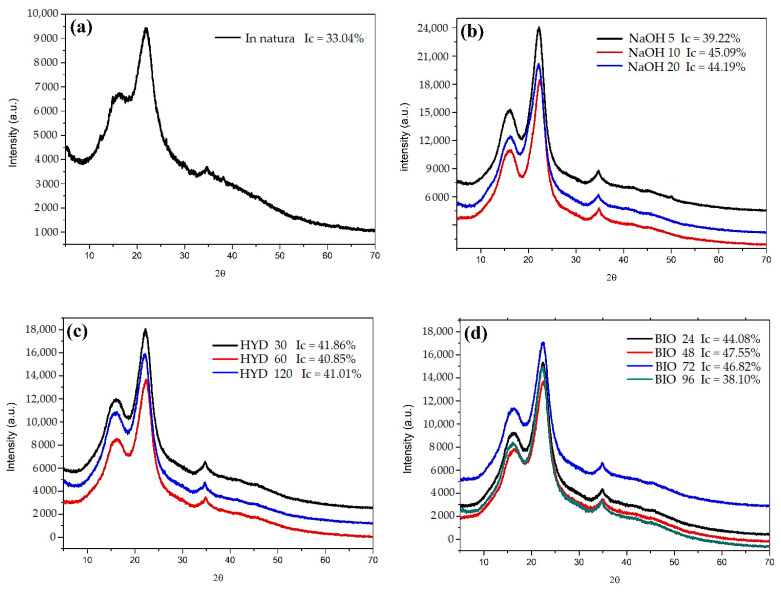
XRD for samples after chemical treatments, (**a**) in natura CHFW; (**b**) NaOH; (**c**) HYDRO; and (**d**) BIO treatment.

The in natura CHFW diffractogram (Figure 4) shows two well-defined peaks. The first of lower intensity at 2θ = 15.02° refers to the crystallographic plane (1 1 0) [28]. A second peak of higher intensity at 2θ = 22.02° (2 0 0) [28,42] identifies a larger fraction of crystalline material for this position. A third, almost imperceptible, peak was observed at 2θ = 34.74° (0 4 0) [43]. The analysis of this peak indicates an ordered structure similar to type I cellulose with an Ic of 33.04% [43,44]. 

Collazo-Bigliardi at al. [28] and Sung et al. [5] reported similar angles for the same crystallographic planes when analyzing coffee husks and endocarp wet processing residues, with Ics of 38 and 49%, respectively. 

Table 3 shows the increased Ics after fiber treatments. These results are in accordance with the lower extractives and hemicellulose values presented for the CHFW lignocellulosic composition (Table 2). NaOH 10 with 45.09%, BIO 48 with 47.55%, and BIO 72 with 46.82% presented the highest rates. The XRD analysis presents some variations, and therefore, the results attributed to NaOH 10 and NaOH 20 can be considered equal. The long exposure time of fibers to the fungus may have degraded the cellulose with lignin, thus decreasing the degree of crystallinity in the BIO 96 treatment.

The removal efficiency of extractive compounds and the greater exposure of cellulose molecules through mercerization treatments were reported by other authors for different types of natural fibers wastes such as banana stalk [31], garlic straw [43], coconut shell [44], parchment in coffee [42], and sugarcane bagasse [30]. Only a few studies performed XRD analyses for fibers treated hydrothermally and with fungi. However, Ajouguim et al. [23] observed Ic changes in relation to the treatment times, reaching 86.69% in 1 h, when treating the Moroccan alpha stem fibers through a hydrothermal process. Youssef et al. [35] reported 70% Ic in fibers from citrus trees when treated with *Aspergillus flavus* fungi. In another study, Ilyas et al. [45] obtained a 76% crystallinity index in sugar palm fiber treated with NaOH. In that work, the fibers were pre-treated with acetic acid and sodium chlorite, then subjected to treatment with NaOH for 7 h.

Table 3 compares the diffraction angles of all treatments performed and shows no changes in the cellulose type of CHFW after treatments. Indeed, type I cellulose remains predominant. Generally, all applied treatments removed part of the amorphous compounds of the CHFW, thereby increasing the fibers’ crystallinity. This positive result is expected to improve the strength and stiffness of the polymer composites produced [30]. Maradini et al. [46] reported increases in the flexural modulus and toughness of polyester resin composites and 2% of cellulose nanocrystals. Sung et al. [5] observed an increase in the crystallinity of biodegradable lactic polyacid films when 3% of cellulose nanocrystals extracted from the endocarp of coffee husks were added, thereby improving mechanical and barrier properties.

### 3.4. SEM

**Figure 5:**
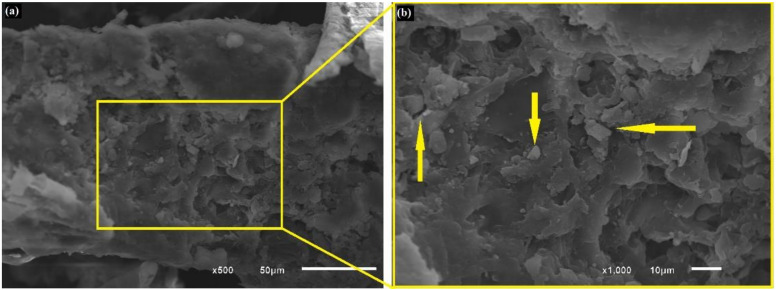
Photomicrographs of fresh in natura CHFW; (**a**) 500× and (**b**) 1000×.

**Figure 6:**
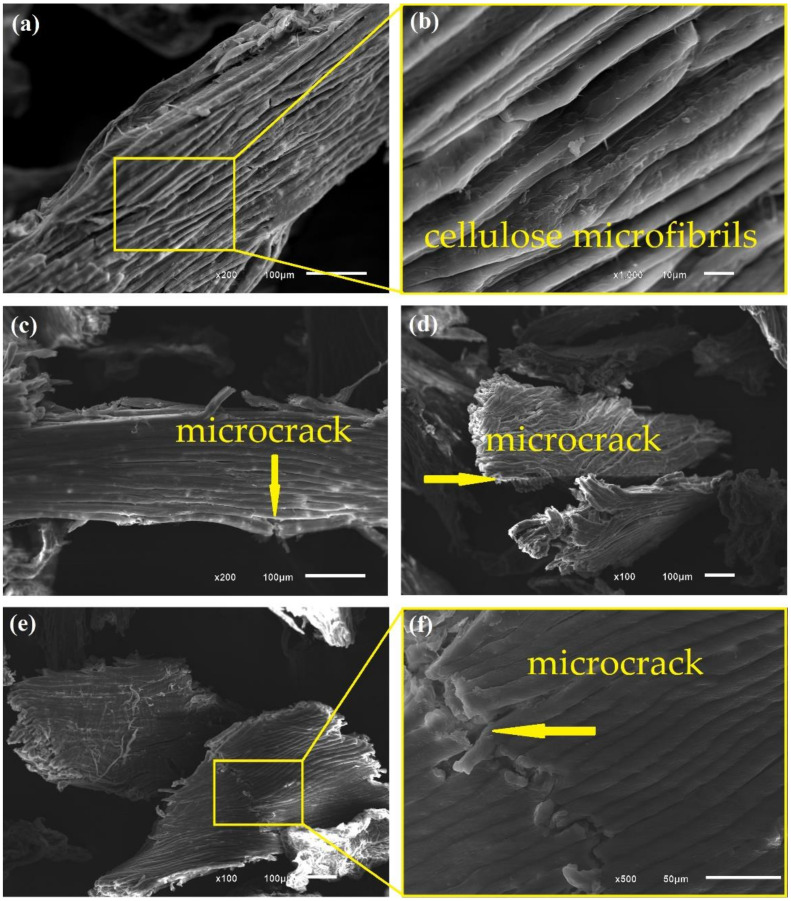
Photomicrographs of CHFW after NaOH 5 (**a**,**b**); NaOH 10 (**c**,**d**); and NaOH 20 (**e**,**f**) treatments.

**Figure 7:**
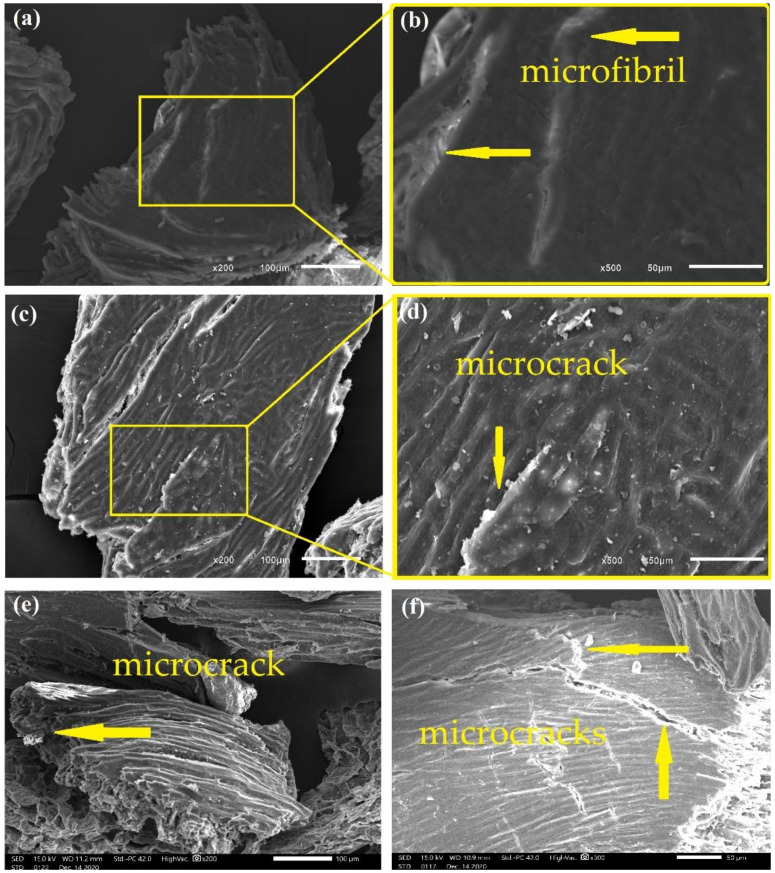
Photomicrographs of CHFW after HYD 30 (**a**,**b**); HYD 60 (**c**,**d**); and HYD 120 (**e**,**f**) treatments.

**Figure 8:**
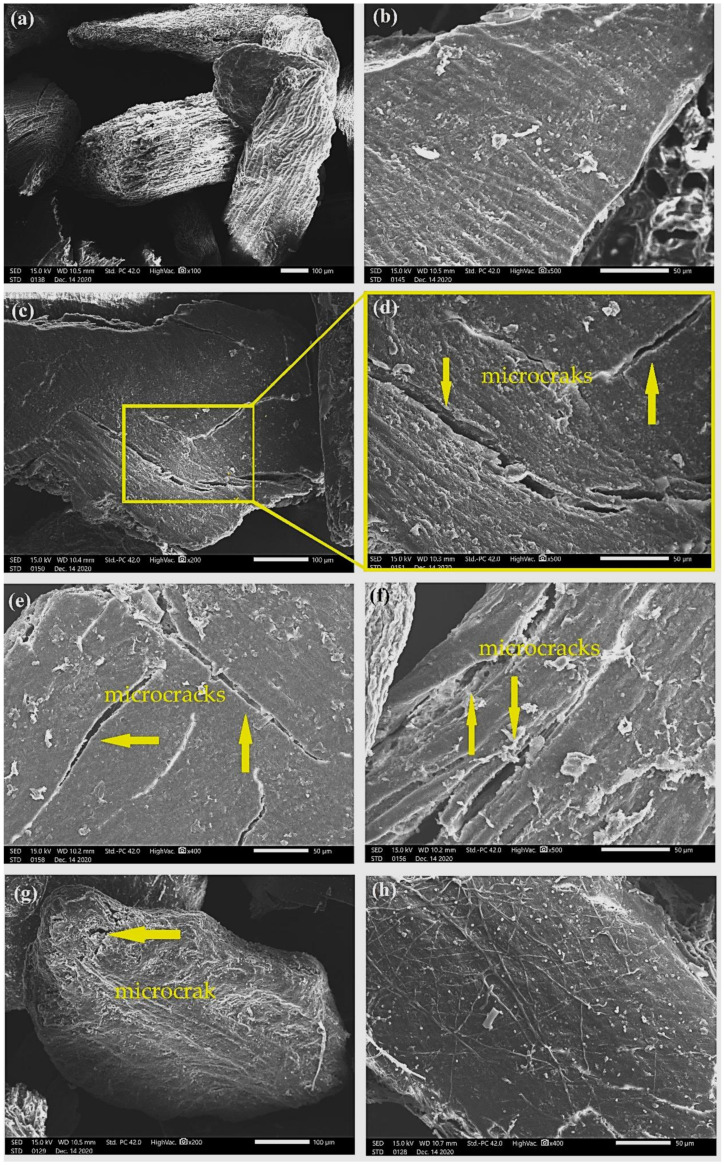
Photomicrographs of CHFW after BIO 24 (**a**,**b**); BIO 48 (**c**,**d**); BIO 72 (**e**,**f**); and BIO 96 (**g**,**h**) treatments.

Figure 5, Figure 6, Figure 7 and Figure 8 show fresh in natura CHFW as well as after chemical, physical, and biological treatment.

The SEM photomicrographs in Figure 5 reveal a material appearance characteristic of lignocellulosic particles with an irregular multilayer surface. Collazo-Bigliardi et al. [28] also made similar observations, indicating that the fibers are CHF particles. In Figure 5, these particles are pointed by arrows and partially covered by the extractive materials.

Figure 6, Figure 7 and Figure 8 show the surfaces after distinct treatments. Mercerization with NaOH produced greater fiber roughness as shown in Figure 6, as and also reported by D’Almeida et al. [47]. Treatment with 5% NaOH solution, Figure 6a,b, provides good exposure of cellulose microfibrils without any material cracking. Therefore, fibers treated with 5% NaOH are more likely to adhere to the matrix, thus improving the composites’ properties. 

An increase in alkaline solution concentration damaged the CHFW surface, as illustrated in Figure 6c–f. Bartos et al. [30] and Kabir et al. [14] also reported the deterioration of the lignocellulosic material associated with higher alkaline concentrations. In fact, higher levels of mercerization in sample NaOH 10, Figure 6c,d, as well as NaOH 20, Figure 6e,f disclose microcracks pointed by arrows. Thus, despite greater delignification with the increase in alkali concentration, there is an ideal concentration and immersion time for the fibers delignification without damaging their properties.

Hydrothermal treatment was also able to promote the exposure of cellulose microfibrils, as shown in Figure 7. Although to a lesser extent, at the exposure for 30 min, HDY 30 in Figure 7a,b disclosed microfibrils without damaging the CHFW particles, as occurred for HYD 60 and 120, reveling microcracks in Figure 7c–f. Similar behavior was found in the biological treatment in which the short time of 24 h, BIO 24, is associated with undamaged CHFW particles, Figure 8a, and a relatively smooth surface, Figure 8b. Longer times, however, promote microcracks, Figure 8c–g, in spite of rather smooth surfaces, as in Figure 8h.

In general, apart from slight roughness, the analysis of the physical and biological treatments in Figure 7 and Figure 8 revealed no major surface changes, which justify the reduced levels of extractives, observed in Table 2. A similar appearance was reported by Qian et al. [48] and Kristensen et al. [39] in hydrothermally treated bamboo and wheat straw fibers, as well as by Jayapriya and Vigneswaran [34] in jute fibers treated with *P. chrysosporium* fungi. 

Small particles distributed on the CHFW surface were observed in the photomicrographs of Figure 8(b,h). These may be associated with traces of extractive material or the formation of pseudolignin, which are substances formed from hemicellulose and lignin waste at low values of pH [49]. In general, hemicellulose and lignin are materials with amorphous characteristics that, owing to increased temperature and pressure, tend to go through a stage of glass transition between 80 and 193 °C. Pretreatments conducted at higher temperatures can coalesce lignin molecules, which migrate from the cell wall and deposit on fiber surfaces [50,51,52,53]. Qian et al. [48], Kristensen et al. [39], and Araya et al. [49] observed this same behavior in bamboo, wheat straw, and eucalyptus fibers, respectively. 

### 3.5. TGA and DTG 

**Figure 9:**
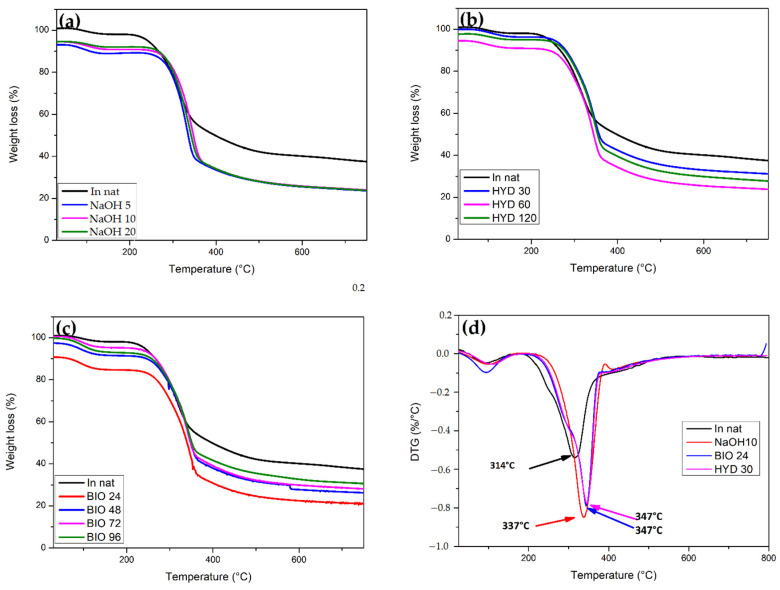
(**a**) TGA NaOH, (**b**) TGA HYD, (**c**) TGA BIO, and (**d**) DTG for fresh CHFW, in natura, as well as subject to NaOH, HYD, and BIO treatment.

The thermal stability of NFLs is one of the limiting factors regarding the use of these materials as fillers in polymer composites. Therefore, knowledge of their degradation properties is crucial in evaluating these materials’ ability to withstand industrial processing temperatures [14]. The thermograms in Figure 9 provide information on the degradation temperatures of fibers constituents in natura CHFW and after treatment. 

The CHFW shows some decomposition steps of its lignocellulosic because of their cell structure degradation, similar to any typical NLF. Being an amorphous material with a lower degree of polymerization and organization, hemicellulose tends to decompose first between 250 and 350 °C. It is followed by cellulose decomposition at 275–450 °C. The latter, despite its higher degree of polymerization, does not have aromatic ramifications in its structure, thereby adding greater stability to the polymer. This is why the lignin gradually decomposes from 250 to 500 °C [26,52]. 

The degradation profiles of the CHFW exhibit mass loss around 5%, Figure 9a, at temperatures below 150 °C, which is attributed to the elimination of water molecules and volatile extractives [54,55]. As the mercerization treatments eliminate hemicellulose molecules, no sharp peaks in TG curves close to 244 °C are noticed, which mark its second stage of decomposition. Residual hemicellulose chains underwent depolymerization at temperatures around 180 °C [51,52,53,54]. Moreover, HYD 30 and BIO 24 treatments, Figure 9b,c, revealed the highest thermal stability for cellulose molecules’ depolymerization, with practically the same maximum degradation temperatures of 347 °C in the DTG curves of Figure 9d. For the chemical treatment NaOH 10, the maximum degradation rate occurred at slightly lower temperature of 337 °C. In contrast, this rate of degradation for in natura CHFW occurred at a sensibly lower 314 °C, as shown in Figure 9d. 

These results may be related to the higher Ic’s in Table 3 of this condition when compared with other treatment conditions, where more compact and rigid structures tend to decompose at higher temperatures [55].

For the HYD 30 treatment, the first stage of degradation started at 103 °C, a value higher than that of the in natura CHFW at 94 °C, indicating that those samples may have absorbed more moisture during treatment.

**Figure 10:**
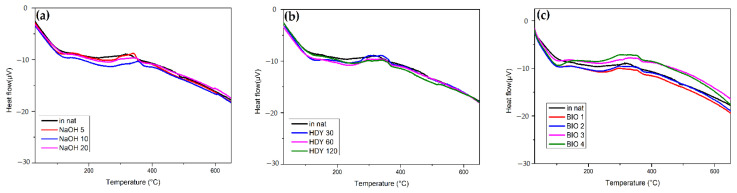
DSC for (**a**) NaOH; (**b**) HDY; and (**c**) BIO treatment.

Figure 10 shows the DSC results of NaOH, HDY, and BIO treatment. In this figure, two exothermic events are observed, the first at 150–200 °C associated with cellulose and hemicellulose degradation. The other events at 300 to 370 °C are attributed to lignin degradation. For all treatments, there are no significant differences between the thermograms.

### 3.6. Preliminary Analysis of Composites Produced Using Treated Fibers

To evaluate the influence of the treatments used on the tensile strength of COPU matrix, biocomposites with 20% CHFW were preliminarily evaluated. Based on the results of the chemical, FTIR, and morphological analyses, one treatment was chosen to represent each of the classes, namely, NaOH 5, HYD 30, and BIO 1, as well as in natura CHFW for the control. These treatments improved the CHFW surface area, as shown in Figure 4a,b, Figure 5a,b and Figure 6a,b. Here, it is worth mentioning that this parameter is important to the natural fiber/polymer matrix interface in composites. The typical natural fibers surface area is associated with the Brunauer–Emmett–Teller (BET) values of 0.4 to 0.5 m^2^·g^−1^ [56].

**Figure 11:**
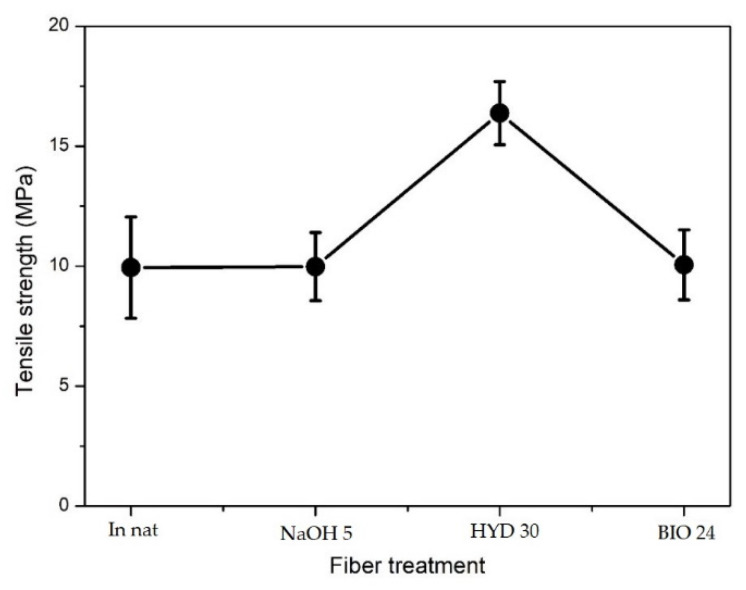
Tensile strength of composites with 20% treated and untreated CHFW.

The tensile strength results of the biocomposites are shown in Table 4 and Figure 11. Biocomposites with 20% of CHFW hydrothermally treated for 30 min (HYD 30) resulted in a tensile strength 60% higher than the other treated CHFW/COPU biocomposites. Moreover, there was no significant difference between the composites with fibers treated with NaOH 5 or BIO 24 in comparation with the untreated in natura CHFW. One can notice on Table 2 that the HYD 30 treatment was not the most effective in exposing the cellulose. On the other hand, it was the most efficient relating to the reduction of extractive material. Based on Figure 11, it is also worth noting that this treatment did not promote high degradation in the CHFW.

**Figure 12:**
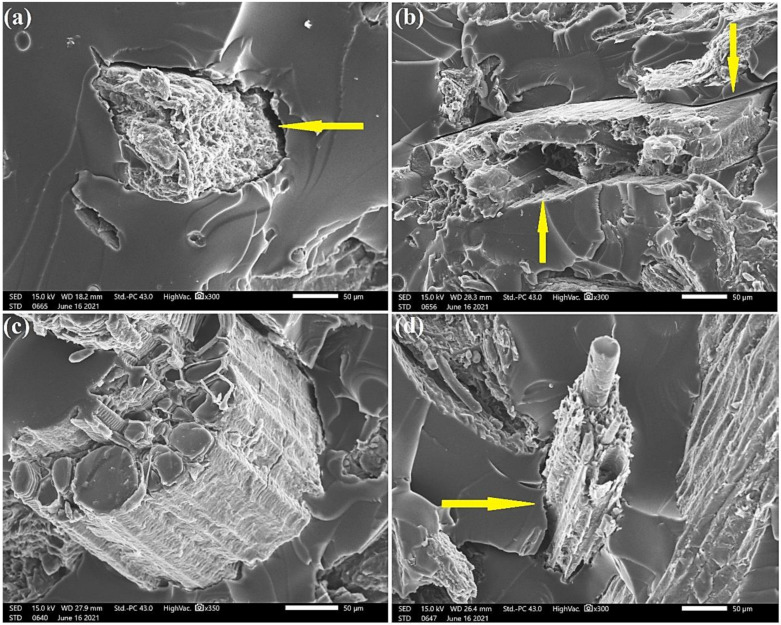
SEM micrographs of the fracture surface of composites with 20% CHFW after the following treatments: (**a**) in natura; (**b**) NaOH 5; (**c**) HYD 30; (**d**) BIO 24.

SEM images in Figure 12 demonstrate the expressive increase in the tensile strength of HYD 30 composites, which can be explained by the better interfacial interaction between the fibers and the COPU matrix. By comparing the interfacial failures by decohesion (arrows) in the composites with 20% in natura, NaOH 5, and BIO 24, the results of the ANOVA and Tukey’s test, shown in Table 5 and Table 6, respectively, prove such a significant increase. This improvement in interfacial bonds is mainly attributed to the reduction in the extractives present in the fibers. These extractives are made of oily and resinous materials that can act as a barrier between the fibers and the polymer matrix.

## 4. Conclusions

Coffee husk fiber waste (CHFW) was chosen as a filler of polymeric biocomposites to improve their mechanical properties given its mass production and the considerable amount of this agricultural residue. The characterization of this CHFW confirms this renewable resource’s potential application for biocomposites. The chemical treatment achieved the exposure of cellulose microfibrils through the elimination of amorphous and low molar compounds. As such, it provides exposure of the cellulose molecules and the dissolution of the hemicellulose molecules. This was confirmed by using FTIR analysis, which revealed absorption bands converging to 2358 cm^−1^. 

The physical treatments by hydrothermal exposure at 120 °C and pressure of 98 KPa were able to expose the cellulose microfibrils after 30 min (HYD 30). However, hydrothermal times of 60 and 120 min promoted damage to the CHFW particles. The biological treatment with *P*. *chrysosporium* fungus promoted the effective exposure of cellulose microfibrils after 24 h (BIO 24), still maintaining relatively smooth CHFW surfaces, and the extractive materials were observed by SEM for exposures after 48, 72, and 96 h. 

The XRD results show an increase of CHFW crystallinity index (I_c_) for all treatment, indicating that this crystalline fraction can improve the composites’ mechanical and thermal properties. However, the SEM photomicrographs reveal that the ideal chemical treatment is for mercerization with 5% NaOH concentration. Microcracks were observed for 10% and 20% concentrations, indicating that the material has been degraded. Increases in the thermal degradation of the CHFW were detected by TGA. The maximum rate values of the degradation associated with DTG peaks for chemical treatment were as follows: NaOH 5, 337 °C; hydrothermal treatment HYD 30, 347 °C; and biological treatment BIO 24 at 347 °C, which are significantly higher than that of in natura CHFW at 314 °C. 

The preliminary tensile test of castor-oil-based polyurethane matrix biocomposites incorporated with 20 wt.% of in natura and different treated CHFW showed that among the optimized condition, the HYD 30 was the only on that resulted in a significant increase: 60% of the tensile strength in comparison to the others. The CHFW treatments and the preliminary tensile result indicate a potential for a low-cost agricultural residue to be incorporated in castor-oil-based polyurethane as sustainable biocomposites, with potential to be applied as plastic wood for household and automobile parts.

## Figures and Tables

**Table 1:** Some treatments of natural fiber reinforced biocomposites. Adapted from Nurazzi et al. [15].

Natural Fiber	Matrix	Method	Mechanical Performance
Alfa fiber	Poly(lactic) acid	Calendaring	Composite material improved 34% of Young’s modulus
Flax fiber	Thermoplastic polyolefin	Corona surface treatment	Surface treatment induces an increase in elongation by 14% and a decrease in tensile strength and Young’s modulus of about 14% and 21%, respectively
Sugar palm yarn fiber	Unsaturated polyester	Yarning process	50 wt.% fiber loading contributed to a decrease in tensile strength by almost 17%, enhanced its tensile modulus by 10%
Alfa fiber	Poly(lactic) acid	0.4 M NaOH alkaline treatment	The tensile strength and Young’s modulus of treated 20 wt.% fiber loading improved by 17% and 45%, respectively
Water hyacinth fibers	Bioepoxy resin	1% solution of (3-aminopropyl) triethoxysilane silane treatment	Tensile modulus and strength of WHF/epoxy improved by 1.1% and 15.7%, respectively
Sugar palm fiber	Polyurethane	2% silane treatment	Tensile strength enhanced by 30%

**Table 2:** Lignocellulosic composition and true density of *fresh* and treated CHFW.

	Cellulose (%)	Hemicellulose (%)	Lignin (%)	Extractives (%)	Ashes (%)	Density (g/cm^3^)
IN NAT (fresh)	30.9 ± 1.9	28.5 ± 2.4	22.2 ± 0.9	18.9 ± 1	5.4 ± 0.5	1.3 ± 0.5
NaOH 5	50.0 ± 1.8	17.6 ± 1	22.5 ± 0.5	9.9 ± 0.5	1.4 ± 0.8	1.2 ± 0.5
NaOH 10	48.9 ± 3.1	14.2 ± 2.7	24.8 ± 1.2	12.1 ± 0.5	1.4 ± 0.5	1.2 ± 0.5
NaOH 20	52.8 ± 3.4	12.0 ± 1.7	25.7 ± 0.9	9.5 ± 0.5	1.5 ± 0.7	1.2 ± 0.5
HYD 30	35.1 ± 0.5	30.3 ± 1.8	25.4 ± 2.2	9.2 ± 0.5	1.6 ± 0.5	1.4 ± 0.5
HYD 60	28.0 ± 2.2	35.6 ± 1.6	24.7 ± 2.2	11.7 ± 0.8	1.7 ± 0.5	1.5 ± 0.5
HYD 120	31.1 ± 2.1	34.2 ± 2.1	23.1 ± 0.5	11.6 ± 0.5	1.3 ± 0.5	1.3 ± 0.5
BIO 24	38.5 ± 1.0	27.1 ± 1.3	25.3 ± 1.7	9.1 ± 05	2.9 ± 0.5	1.2 ± 0.5
BIO 48	38.8 ± 2.9	27.2 ± 1.4	24.3 ± 0.5	9.7 ± 0.5	2.1 ± 0.5	1.2 ± 0.5
BIO 72	36.9 ± 1.1	26.9 ± 0.7	25.7 ± 1.2	10.5 ± 0.8	2.3 ± 0.5	1.2 ± 0.5
BIO 96	36.7 ± 0.5	26.4 ± 0.5	26.2 ± 1.0	10.7 ± 0.5	3.2 ± 0.5	1.2 ± 0.5

IN NAT = in natura; HYD 30, HYD 60, and HYD 120 = hydrothermal treatments; NaOH 5, NaOH 10, and NaOH 20 = chemical treatments; BIO 24, BIO 48, BIO 72, and BIO 96 = biological treatments.

**Table 3:** Crystallinity index of in natura CHFW and after treatments.

Treatment	Ic (%)
IN NAT (fresh)	33.04
NaOH 5	39.22
NaOH 10	45.09
NaOH 20	44.19
HYD 30	41.86
HYD 60	40.85
HYD 120	41.01
BIO 24	44.08
BIO 48	47.55
BIO 72	46.82
BIO 96	38.10

**Table 4:** Results of tensile strength of composites with 20% treated and untreated CHFW.

Treatment	Tensile Strength (MPa)
IN NAT	9.9 ± 2
NaOH 5	9.8 ± 1
HDY 30	16.3 ± 1
BIO 24	10.1 ± 1

**Table 5:** Variance analysis of average elastic modulus obtained for tensile strength of composites with 20% treated and untreated CHFW.

	Sum of Squares	df	Mean Square	F	*p* (same)
Between Groups:	155.424	3	51.8079	19.97	0.0000117
Within Groups:	41.5169	16	2.59481		
Total:	196.941	19			

**Table 6:** Results obtained from the Tukey’s pairwise comparisons (Q/p) between the average values of the tensile strength of composites with 20% treated and untreated CHFW.

	IN NAT	NaOH 5	HYD 30	BIO 24
In Nat		0.9997	0.000225	0.9995
NaOH 5	0.1396		0.000218	0.9964
HYD 30	8.942	9.082		0.000243
BIO 24	0.1658	0.3054	8.776	

## Data Availability

The data presented in this study are available upon request to the corresponding author.
